# Does Combining Biomarkers and Brain Images Provide Improved Prognostic Predictive Performance for Out-Of-Hospital Cardiac Arrest Survivors before Target Temperature Management?

**DOI:** 10.3390/jcm9030744

**Published:** 2020-03-10

**Authors:** Seung Ha Son, In Ho Lee, Jung Soo Park, In Sool Yoo, Seung Whan Kim, Jin Woong Lee, Seung Ryu, Yeonho You, Jin Hong Min, Yong Chul Cho, Won Joon Jeong, Se Kwang Oh, Sung Uk Cho, Hong Joon Ahn, Changshin Kang, Dong Hun Lee, Byung Kook Lee, Chun Song Youn

**Affiliations:** 1 Department of Emergency Medicine, Chungnam National University Hospital, 282, Munhwa-ro, Jung-gu, Daejeon 35015, Korea; mpleplem@cnuh.co.kr (S.H.S.); mdinsool@cnuh.co.kr (I.S.Y.); emfire@cnuh.co.kr (S.W.K.); emd93@cnuh.co.kr (J.W.L.); rs0505@naver.com (S.R.); yyh1003@hanmail.net (Y.Y.); shiphid@hanmail.net (J.H.M.); boxter73@naver.com (Y.C.C.); gardenjun@hanmail.net (W.J.J.); 13744@hanmail.net (S.K.O.); mp5medical@naver.com (S.U.C.); jooniahn@hanmail.net (H.J.A.); changsiny@naver.com (C.K.); 2 Department of Radiology, College of Medicine, Chungnam National University, 266, Munhwa-ro, Jung-gu, Daejeon 35015, Korea; leeinho1974@hanmail.net; 3 Department of Emergency Medicine, College of Medicine, Chungnam National University, Daejeon 35015, Korea; 4 Department of Emergency Medicine, Chonnam National University Medical School, Gwangju 61469, Korea; ggodhkekf@hanmail.net (D.H.L.); bbukkuk@hanmail.net (B.K.L.); 5 Department of Emergency Medicine, Seoul St. Mary’s Hospital, The Catholic University of Korea, Seoul 06591, Korea; ycs1005@catholic.ac.kr

**Keywords:** out-of-hospital cardiac arrest, prognosis, neuron-specific enolase, magnetic resonance imaging, computed tomography

## Abstract

We examined whether combining biomarkers measurements and brain images early after the return of spontaneous circulation improves prognostic performance compared with the use of either biomarkers or brain images for patients with cardiac arrest following target temperature management (TTM). This retrospective observational study involved comatose out-of-hospital cardiac arrest survivors. We analyzed neuron-specific enolase levels in serum (NSE) or cerebrospinal fluid (CSF), grey-to-white matter ratio by brain computed tomography, presence of high signal intensity (HSI) in diffusion-weighted imaging (DWI), and voxel-based apparent diffusion coefficient (ADC). Of the 58 patients, 33 (56.9%) had poor neurologic outcomes. CSF NSE levels showed better prognostic performance (area under the curve (AUC) 0.873, 95% confidence interval (CI) 0.749–0.950) than serum NSE levels (AUC 0.792, 95% CI 0.644–0.888). HSI in DWI showed the best prognostic performance (AUC 0.833, 95% CI 0.711–0.919). Combining CSF NSE levels and HSI in DWI had better prognostic performance (AUC 0.925, 95% CI 0.813–0.981) than each individual method, followed by the combination of serum NSE levels and HSI on DWI and that of CSF NSE levels and the percentage of voxels of ADC (AUC 0.901, 95% CI 0.792–0.965; AUC 0.849, 95% CI 0.717–0.935, respectively). Combining CSF/serum NSE levels and HSI in DWI before TTM improved the prognostic performance compared to either each individual method or other combinations.

## 1. Introduction

Despite recent advances in emergency medicine and resuscitation management such as target temperature management (TTM), only approximately 30% of cardiac arrest (CA) survivors are discharged with a good cerebral performance status [1].

Current guidelines recommend determining the neurologic prognosis at 72 h post-CA; however, the withdrawal of life-sustaining treatment (WLST) earlier than 72 h has been reported to be common and was shown to increase the mortality rate among patients who survived CA [2,3]. Furthermore, one study suggested that 26% of patients with an early WLST may have survived and that 64% of these patients may have had a functionally favorable outcome [3]. Moreover, WLST occurred most frequently within 1 day of the return of spontaneous circulation (ROSC) [3]. Therefore, to avoid sub-optimal WLST, an early and accurate prognosis is necessary.

Several prognostic methods have been evaluated for predicting neurologic outcome [2,4]. However, sedatives have been shown to confuse outcome predictions of CA survivors during neurologic examination [5]. Moreover, as a diagnostic tool, an electroencephalogram is subjective and laborious, with the results depending on an interpreter’s expertise [6]. A somatosensory evoked potential recording requires appropriate skills and experience, and it has been reported that artefacts significantly affect the results [4]; however, brain imaging results and biomarkers levels do not exhibit such effects.

Recently, combinations of different prognostic predictive methods have been tested to improve prognostic performance [7,8,9]. However, which of these combinations is most accurate for prognostic prediction remains unclear.

We previously reported that neuron-specific enolase in cerebrospinal fluid (CSF NSE) predicts the prognosis of patients with CA [10] and we reported that results of brain computed tomography (CT) and magnetic resonance imaging (MRI) prior to TTM can be used to predict prognosis in CA survivors [8]. In this study, we hypothesized that combining the NSE level and brain images of CA survivors who had undergone TTM immediately after ROSC may have better prognostic performance than any single analysis.

## 2. Experimental Section

### 2.1. Study Design and Population

**Figure 1:**
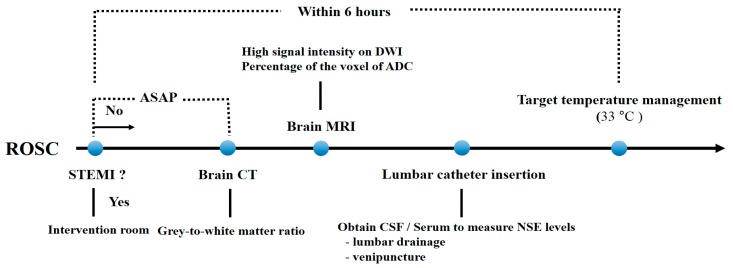
Timeline of examinations and procedures for patients included in this study. Abbreviations: ROSC, return of spontaneous circulation; STEMI, ST-elevation myocardial infarction; ASAP, as soon as possible; CT, computed tomography; MRI, magnetic resonance image; CSF, cerebrospinal fluid; NSE, neuron-specific enolase; DWI, diffusion-weighted imaging; ADC, apparent diffusion coefficient.

This was a retrospective analysis of prospectively collected data including adult comatose out-of-hospital cardiac arrest (OHCA) survivors treated with TTM at Chungnam National University Hospital (CNUH) in Daejeon, Korea, between May 2018 and August 2019. Data acquisition and analysis was performed in compliance with protocols approved by the Ethical Committee of CNUH (ethical approval number 2019-12-013). Written informed consent was obtained from all participants prior to study. We included patients with collected NSE (serum, CSF, or both) levels who underwent brain imaging (CT, MRI, or both) after ROSC (Figure 1). Exclusion criteria were as follows: (1) patients aged <18 years; (2) patients with traumatic CA; (3) patients with an interrupted TTM (because of transfer from another facility or hemodynamic instability (less than 60 mmHg mean arterial pressure or less than 90 mmHg systolic blood pressure even after 6 h or more of the vasopressor support)); (4) patients not eligible for TTM (i.e., because of intracranial hemorrhage, active bleeding, a known terminal illness, or a poor pre-CA neurologic status); (5) patients administered extracorporeal membrane oxygenation; and (6) patients ineligible for lumbar puncture (LP) (i.e., brain CT showed severe cerebral edema, obliteration of the basal cisterns, or an occult intracranial mass lesion).

### 2.2. TTM Protocol

The patients were managed according to our previously published TTM protocol [9]. A target temperature was maintained at 33 °C for 24 h using feedback-controlled surface-cooling devices (Artic Sun^®^ Energy Transfer Pads™; Medivance Corp, Louisville, CO, USA). Midazolam (0.05 mg/kg intravenous bolus, followed by a titrated intravenous continuous infusion of 0.05–0.2 mg/kg/h) and cisatracurium (0.15 mg/kg intravenous bolus, followed with an infusion of up to 0.3 mg/kg/h) were administered for sedation and to control shivering. All patients were treated with standard intensive care according to our institutional intensive care unit protocol.

### 2.3. Measurement of NSE Levels in CSF and Serum

Serum and CSF NSE levels were measured between brain imaging and initiation of TTM. CSF was obtained via lumbar catheter drainage, and serum was collected by venipuncture at the same time. To measure the NSE level, an electrochemiluminescence immunoassay kit (COBAS^®^ e801, Roche Diagnostics, Basel, Switzerland) was used. The NSE measurement range was 0.1–300 ng/mL (normal value, <16.3 ng/mL).

### 2.4. Grey-To-White Matter Ratio Measurement Using Brain CT

Our TTM protocol recommends obtaining a brain CT as soon as possible after ROSC, but this is not mandatory. CT was performed with a 64-channel system (Somatom Sensation 64, Siemens Healthineers, Munich, Germany). A neuroradiologist who was blinded to clinical outcome measured in Hounsfield units (HU) calculated the grey-to-white matter ratio (GWR). HU were recorded at the caudate nucleus (CN), posterior limb of the internal capsule (PIC), corpus callosum (CC), putamen (P), and thalamus (T). GWRcc was defined as the mean value of the ratios CN/CC, P/CC, and T/CC, and the average GWR was defined as the mean value of the 6 ratios CN/CC, P/CC, T/CC, CN/PIC, P/PIC, and T/PIC.

### 2.5. MRI (High Signal Intensity in DWI and Percentage of Voxels of ADC)

MRI was performed using a 3T scanner (Achieva, Philips Healthcare, Amsterdam, The Netherlands) before TTM. Forty continuous DWI sections per patient were acquired using a standard *b* = 1000 s/mm^2^. All images were assessed by a board-certified neuroradiologist who was blinded to the clinical information. The MRI was set to positive when there was high signal intensity (HSI) in DWI, regardless of the volume and location following ischemic injury.

For quantitative analysis of ADC, images were processed and analyzed using a software (FMRIB Software Library, Release 5.0 (c) 2012, The University of Oxford) which can extract brain tissue images by eliminating those of cranium, optic structures, and soft tissues outside the cranium. Images were retrieved in a Digital Image and Communications in Medicine format from picture archiving and communication system servers at the hospital and converted to NITFI format using MRIcron (http://www.nitrc.org/projects/mricron). Voxels with ADC values above 1590 × 10^−6^ mm^2^/s were extracted from the analysis to exclude artefacts or pure CSF. Previous studies have used the percentage of voxels (PV) below different ADC thresholds and reported that the PV 400 (percentage of voxels below 400 × 10^−6^ mm^2^/s) showed the highest odds ratio value when predicting the poor prognosis of a patient; therefore, we also used the PV 400 [10].

### 2.6. Outcome

The primary endpoint of this study was neurologic outcome at 3 months after CA. We measured neurological outcomes 3 months after ROSC using the Glasgow Pittsburgh CPC scale, through either face-to-face interviews or structured telephone interviews [11]. Phone interviews were undertaken by an emergency physician who was fully informed of the protocol and blinded to the patient’s prognosis and NSE levels. Neurologic outcome was assessed using the Glasgow–Pittsburgh Cerebral Performance Categories (CPC) scale and recorded as CPC 1 (good performance), CPC 2 (moderate disability), CPC 3 (severe disability), CPC 4 (vegetative state), or CPC 5 (brain death or death) [12]. A good neurologic outcome was defined as a CPC of 1 or 2, and a poor outcome was defined as a CPC of 3, 4, or 5.

### 2.7. Data Collection

The following data were obtained from electronic medical records: age, sex, first monitored rhythm, etiology of CA, presence of a witness at the time of collapse, bystander cardiopulmonary resuscitation, low flow time, no flow time, time from ROSC to brain image and lumbar puncture, and neurologic outcome at 3 months after CA.

### 2.8. Data Analysis

Continuous variables were reported as the median and interquartile range, as all continuous variables showed a non-normal distribution. Categorical variables were compared using the chi-square test. The receiver operating characteristic (ROC) was analyzed with the corresponding area under the curve (AUC) with the 95% confidence interval (CI). Cut-off values with 100% specificity were calculated for predicting poor neurologic outcomes at 3 months after CA. The AUC values of the combinations were estimated in two steps. First, a probability value was obtained by binary logistic regression analysis. Second, we conducted ROC analysis using this probability as a test variable. Data were analyzed using SPSS for Windows, version 18 (SPSS, Inc., Chicago, IL, USA). ROC curves were calculated and compared using MedCalc version 14.8.1.0 (MedCalc Software, Mariakerke, Belgium) software. Statistical significance was set to *p* < 0.05.

## 3. Results

### 3.1. Patient Demographics

**Figure 2:**
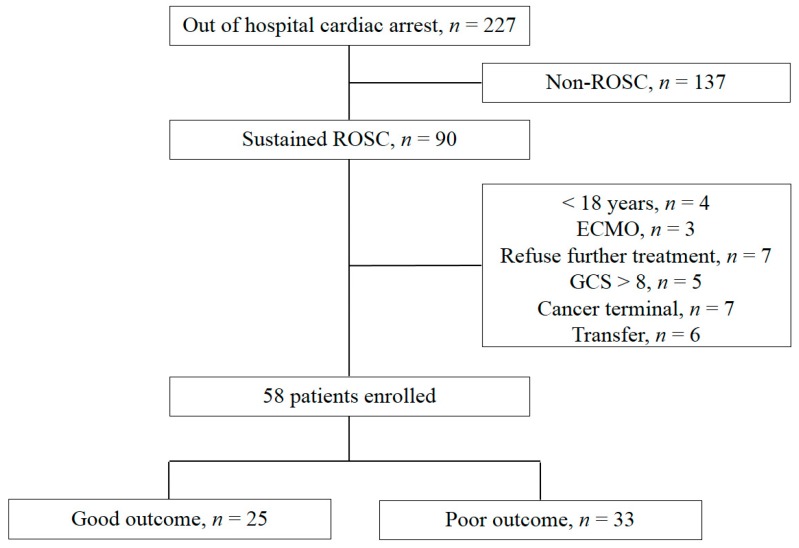
Flow diagram of patient selection. Abbreviations: ROSC, return of spontaneous circulation; ECMO, extracorporeal membrane oxygenation; GCS, Glasgow coma scale.

Of a total of 227 patients with OHCA who had visited the emergency medical center, 137 had no sustained ROSC. The following patients were excluded: four were <18 years old; three were treated with extracorporeal membrane oxygenation; the families of seven patients declined offers of further treatment, five had a Glasgow coma scale (GCS) score >8; seven were patients with terminal cancer; six patients were transferred to another facility. Finally, 58 patients were included in this study, and 33 (56.9%) had poor neurologic outcomes (Figure 2). Baseline demographic and clinical characteristics are presented in Table 1. Serum and CSF NSE levels were obtained in 57 and 50 patients, respectively. Brain CT and MRI were performed in 58 and 57 patients, respectively.

### 3.2. Comparison of Neurologic Outcome Using Each Method

Serum NSE and CSF NSE levels were significantly higher in the poor neurologic outcome group than in the good neurologic outcome group (Table 2, *p* < 0.001, both). The GWR was significantly lower in the poor neurologic outcome group (*p* = 0.005). The poor neurologic outcome group had a positive HSI in 22 (66.7%) patients, whereas the good neurologic outcome group showed no positive HSI (Table 2, *p* < 0.001). The PV 400 of ADC was significantly higher in the poor neurologic outcome group (Table 2, *p* < 0.001).

### 3.3. Prognostic Performance of Each Method

**Figure 3:**
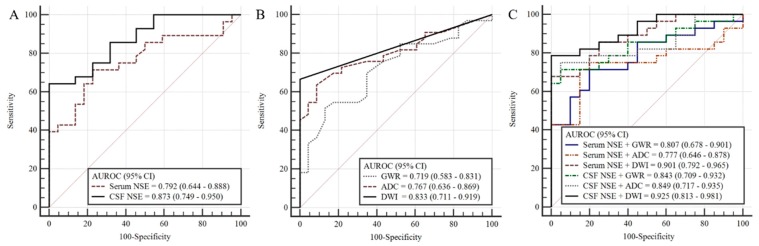
Comparison of receiver operating characteristic curves of (**A**) CSF NSE and serum NSE levels; (**B**) HSI in DWI, voxels of ADC, and GWR in CT, and; (**C**) combinations of NSE levels and brain images. AUROC, area under the receiver operating characteristic curve; CI, confidence interval; HIS: high signal intensity; GWR, grey-to-white matter ratio.

A higher AUC value was found for CSF NSE levels than for serum NSE levels (0.873 (95% CI 0.749–0.950) versus 0.792 (95% CI 0.664–0.888)), respectively (Table 3 and Figure 3A). The CSF NSE and serum NSE sensitivity for predicting poor neurologic outcomes with a specificity of 100% were 46.9% and 64.3%, respectively (Table 3). HSI in DWI showed the strongest prognostic performance with an AUC value of 0.833 (95% CI 0.711–0.919), followed by PV of ADC (AUC 0.767, 95% CI 0.636–0.869) and GWRcc (AUC 0.719, 95% CI 0.583–0.831) (Table 3 and Figure 3B). GWRcc showed higher AUC value for predicting poor neurologic outcome than the average GWR (Table 4).

### 3.4. Prognostic Performance of Combining NSE Levels and Brain Imaging 

In predicting a poor neurologic outcome prognosis, combining CSF NSE levels and HSI in DWI showed the strongest prognostic performance (AUC 0.925, 95% CI 0.813–0.981), followed by, in order, combining serum NSE levels and HSI in DWI (AUC 0.901, 95% CI 0.792–0.965), combining CSF NSE levels and GWR (AUC 0.855, 95% CI 0.724–0.940), combining CSF NSE levels and PV of ADC (AUC 0.849, 95% CI 0.717–0.935), and combining serum NSE levels and GWR (AUC 0.807, 95% CI 0.678–0.901). Combining serum NSE levels and PV of ADC showed the lowest prognostic performance (AUC 0.777, 95% CI 0.646–0.878) (Figure 3C).

## 4. Discussion

In this retrospective observational study, CSF NSE levels showed better predicting prognostic performance than serum NSE levels, and brain imaging effectively predicted prognostic performance in the order of HSI on DWI, PV of ADC, and GWR on CT. Combining NSE levels and brain imaging showed better prognostic performance for predicting poor neurologic outcomes with 100% specificity in CA survivors who were comatose and treated with TTM. The highest prognostic predictive performance combination was CSF NSE levels and HSI in DWI.

This study was performed to determine the optimal combination of analyses for predicting the prognosis of patients with CA early after ROSC. Among the various prognostic predictive methods available, we used NSE (serum or CSF) levels and brain imaging (CT or MRI), as these methods are less susceptible to inter-rater variability and less affected by sedatives than other methods such as clinical examination, electroencephalogram, and somatosensory evoked potential [5,6,13].

Brain CT is a relatively easy, safe, and inexpensive method for predicting prognosis compared to MRI. The 2015 American Heart Association recommends using GWR in CT to predict poor neurologic outcomes [2]. However, a recent prospective multi-center study of 512 patients reported that the GWR assessed via early brain CT alone was not an independent factor predictive of poor neurologic outcomes [14]. Thus, they suggested that brain CT scans performed during the first 2 h after ROSC may not allow sufficient time for the formation of cerebral edema and increased intracranial pressure. In our study, the median time from ROSC to CT scan was 79.0 min (range 43.0–129.0 min), and the prognostic predictive performance was the lowest among the brain imaging methods evaluated. Moreover, this method has low sensitivity, and drawbacks of significant deviation depending on the interpreter [8]. However, MRI has been shown to predict hypoxic–ischemic brain injury (HIBI), proving that its CA prediction ability of comatose survivors after ROSC is superior to that of CT [8,10,15]. Cortical laminar necrosis of the brain may be identified within hours of the anoxic–ischemic event, and MR-DWI offers good visualization of laminar necrosis and other characteristic signs of hypoxic injury with unsurpassed promptness [15]. Cerebral cortical diffusion abnormalities are known to be associated with poor neurologic outcome after CA [15,16]. Furthermore, cytotoxic edema (CytE) occurs in the cortex within minutes of onset of HIBI. When CytE occurs, ADC values are lowered because of the parallel shrinkage of the neuronal cell bodies. A decrease in ADC values may serve as an indication that the affected areas of the brain will die [17]. The severity of CytE represented by lowered ADC values can be measured quantitatively [10,17].

A prognostic predictive method using MRI prior to TTM has been reported using qualitative and quantitative analyses [18]. However, there is no universally accepted standard DWI technique. As a qualitative method, we used the presence of abnormal HSI in DWI. Recent studies showed that MRI can predict neurologic outcomes as early as 3 h after ROSC [8,13,15]. Several animal experiments have shown that HSI in DWI can be observed within 1 h of blood flow loss [19,20]. Therefore, brain damage in patients with CA can be identified earlier using MRI compared to using CT. Jeon et al. reported that the AUC and sensitivity for predicting poor neurologic outcomes in the presence of abnormal HSI in DWI immediately after ROSC (DWI was performed in 175 min (range 117–240] of median time after ROSC) were 0.894 (95% CI 0.753–0.969) and 78.8% (95% CI 61.1–91.0) with 100% specificity, respectively [8]. The quantitative method involves restricted diffusion using cytotoxic edema, which is quantified by calculating the ADC value of each voxel [10,21]. Moon et al. reported that the AUC and sensitivity for poor neurologic outcome prediction of voxels with ADC values under 400 × 10^−6^ mm^2^/s in MR-DWI performed within 48 h after CA were 0.891 (95% CI 0.792–0.989) and 64.0% (95% CI 42.5–82.0) with 100% specificity, respectively [10]. In the present study, qualitative analysis using DWI (HSI) showed more accurate prognostic predictive performance and sensitivity early after CA than quantitative analysis using PV 400. This issue requires further analysis to determine the exact ADC thresholds to be used in predicting neurologic outcomes; an easy way to use the voxels of ADC and CSF contamination should also be considered. In the ADC, the exact division between micro-CSF and brain parenchyma is not specified, and the boundary values are mixed. This is referred to as CSF contamination [22,23].

Vondrakova et al. reported that the AUC of serum NSE levels on the first morning of hospitalization (6–30 h from collapse) for prognosis prediction of poor neurologic outcomes at 1 month was 0.768 (sensitivity 63.3%; specificity 82.1%) [24]. In this study, our results showed that the AUC of serum NSE levels early after ROSC was 0.792 (sensitivity 46.9%; specificity 100%). In a previous study, we reported that CSF NSE levels were more accurate than serum NSE levels for prognostic prediction of poor neurologic outcomes [7]. There is a reasonable explanation for this superior performance. NSE is also found in red blood cells and platelets, and CA survivors commonly have hemolysis, which increases NSE levels without necessarily reflecting ischemic/reperfusion brain injury [25]. In contrast, there are no red blood cells or platelets present in a normal CSF sample. As a result, the measured NSE levels in the CSF of CA survivors were more accurate than those determined in the serum [7].

International guidelines suggest using a multimodal prognostication rather than a single modality for poor neurologic outcome prediction [2,4]. Lee et al. reported that the combination of GWR measured by brain CT immediately after ROSC and serum NSE values measured 48 h after ROSC improved prognostic performance for predicting poor neurologic outcomes [26]. They did not provide accurate AUC values; however, the sensitivity and specificity were 78.6% (95% CI 67.6–86.6%) and 100%, respectively. In our study, a combination of NSE (serum or CSF) levels and brain imaging obtained immediately after ROSC improved prognostic performance compared to using either method alone in predicting poor neurologic outcomes in CA survivors who were comatose. The most powerful predictor of poor neurologic outcome was a combination of CSF NSE levels and HSI in DWI. The AUC value and sensitivity with 100% specificity were 0.925 (95% CI 0.813–0.981) and 77.78%, respectively. Although CSF NSE levels and HSI in DWI were measured early after ROSC, our study results showed more improved performance than that reported by Lee et al. [26]. This combination appears to have a superior performance for predicting poor neurologic outcomes than using any other single method immediately after ROSC. However, HSI identification through CSF NSE measurements and MRI tests has the disadvantage of being very elaborate and costly.

This study has several limitations. First, this was a single-center retrospective study with a small number of patients; therefore, a multicenter prospective study is required to enhance the generalizability of the findings. Second, patients with brain CT, MR-DWI, and/or CSF or serum NSE measurements were included, and combinations of brain CT, MR-DWI, MR-ADC, CSF or serum NSE levels were evaluated. However, LP is rare in clinical practice and is very complex to apply, and MRI costs are high; hence, they are generally not applicable. Third, bias due to self-fulfilling prophecy is possible as the treating physicians were exposed to the results of brain CT, MR-DWI, and CSF or serum NSE measurements. However, WLST was not permitted in Korea prior to February 2018, unless a patient had been pronounced brain-dead. Even since February 2018, WLST has been performed rarely and in a very conservative manner. In this study, no patients underwent WLST during TTM.

## 5. Conclusions

A combination of CSF NSE levels and HSI in DWI can be used to predict poor neurologic outcomes in OHCA survivors who are comatose and treated with TTM early after ROSC. These combinations showed better performance than other combinations or using each method alone. However, LP is not common in the clinical practice for cardiac arrest survivors. We therefore suggest that the combination of serum NSE levels and HSI in DWI routinely performed in clinical practice may also be useful. In addition, the combination of CSF NSE levels and HSI in DWI has the limitation of being very elaborate and expensive. A large sample, multi-center study is needed to identify the precise association between these combined methods and neurologic outcomes.

## Figures and Tables

**Table 1:** Baseline demographics and clinical characteristics.

Characteristics	Cohort (*n* = 58)	Good Outcome (*n* = 25)	Poor Outcome (*n* = 33)	*p*-Value
Age, years, median (IQR)	53.5 (37.6–69.0)	50.5 (43.0–58.1)	55.3 (48.8–61.7)	0.347
Sex, male, *n* (%)	40 (69.0)	20 (80.0)	20 (75.8)	0.155
Charlson Comorbidity Index score, median (IQR)	0.0 (0.0–2.0)	0.0 (0.0–2.0)	0.0 (0.0–1.50)	0.975
Arrest characteristics				
Witness arrest, *n* (%)	36 (62.1)	21 (84.0)	15 (45.5)	0.003
Bystander CPR, *n* (%)	41 (70.7)	21 (84.0)	20 (62.5)	0.085
Shockable rhythm, *n* (%)	19 (33.3)	16 (64.0)	3 (9.4)	0.000
Cardiac aetiology, *n* (%)	17 (30.4)	13 (52.0)	4 (12.9)	0.002
No flow time, min (IQR)	3.5 (0.0–16.0)	0.0 (0.0–5.0)	12.0 (1.0–42.0)	0.002
Low flow time, min (IQR)	20.0 (9.0–33.0)	9.0 (5.5–16.5)	30.0 (19.5–42.5)	<0.001
ROSC to CT time, min (IQR)	79.0 (43.0–129.0)	77.0 (40.5–106.5)	95.0 (43.0–152.0)	0.271
ROSC to MRI time, min (IQR)	180.5 (128.0–240.8)	154.0 (113.5–286.5)	194.0 (129.5–288.5)	0.713
ROSC to LP time, min (IQR)	256.5 (223.8–364.8.0)	239.0 (193.0–430.0)	272.0 (229.0–334.0)	0.303

IQR, interquartile range; CPR, cardiopulmonary resusciation; ROSC, return of spontaneous circulation; CT, computed tomography; MRI, magnetic resonance image; LP, lumbar puncture.

**Table 2:** NSE levels of serum and cerebrospinal fluid (CSF), grey-to-white matter ratio (GWR), high signal intensity (HSI) in DWI, and percentage of voxels (PV) 400 ** of ADC.

Characteristics	Good Neurologic Outcome (*n* = 25)	Poor Neurologic Outcome (*n* = 33)	*p-*Value
Serum NSE, median (IQR), 57 *	26.1 (19.4–33.3), 25 *	48.1 (30.0–90.2), 32 *	<0.001
CSF NSE, median (IQR), 51 *	19.1 (11.8–33.2), 23 *	94.7 (19.2–183.8), 28 *	<0.001
GWR, median (IQR), 58 *	1.24 (1.19–1.29), 25 *	1.16 (1.11–1.24), 33 *	0.005
HSI on DWI, number (%), 57 *	0 (0.0%), 24 *	22 (66.7%), 33 *	<0.001
PV 400 ** on ADC, median (IQR), 57 *	2.28 (0.32–2.93), 24 *	3.90 (2.24–29.24), 33 *	<0.001

NSE, neuron-specific enolase; DWI, diffusion-weighted imaging; ADC, apparent diffusion coefficient. *, Number of patients included in the analysis; **, percentage of voxels below 400 × 10^−6^ mm^2^/s.

**Table 3:** Prognostic performances of levels of serum and CSF NSE, DWI, ADC, and GWR for predicting three-month poor neurological outcome.

Characteristics	AUC (95% CI)	*p*-Value	Cut-Off	Sensitivity/Specificity (%)	PPV	NPV	TP	TN	FP	FN
Serum NSE, 57 *	0.792 (0.664–0.888)	<0.001	54.8	46.9/100	100.0	59.5	15	26	0	16
CSF NSE, 51 *	0.873 (0.749–0.950)	<0.001	53.7	64.3/100	100.0	68.7	18	23	0	10
DWI (HSI), 57 *	0.833 (0.711–0.919)	<0.001	HSI positive	66.7/100	100.0	68.6	22	24	0	11
ADC (PV 400 **), 57 *	0.767 (0.636–0.869)	<0.001	4.3	45.5/100	100.0	57.1	15	24	0	18
GWR, 58 *	0.719 (0.583–0.831)	0.002	1.07	18.2/100	100.0	46.0	6	26	0	26
DWI + Serum NSE, 56 *	0.901 (0.792–0.965)	<0.001		71.9/100	100.0	72.7	23	24	0	9
DWI + CSF NSE, 49 *	0.925 (0.813–0.981)	<0.001		77.8/100	100.0	72.7	22	21	0	6
ADC + Serum NSE, 56 *	0.777 (0.646–0.878)	<0.001		78.6/100	100.0	77.8	16	24	0	16
ADC + CSF NSE, 49 *	0.849 (0.717–0.935)	<0.001		67.9/100	100.0	70.0	18	21	0	10
GWR + Serum NSE, 56 *	0.807 (0.678–0.901)	<0.001		50.0/100	100.0	59.0	16	24	0	16
GWR + CSF NSE, 49 *	0.855 (0.724–0.940)	<0.001		64.3/100	100.0	48.8	18	21	0	10

AUC, area under the curve; CI, confidence interval; PPV, positive present value; NPV, negative present value; TP, true positive; TN, true negative; FP, false positive; FN, false negative; *, number of patients included in the analysis; **, percentage of voxels below 400 × 10^−6^ mm^2^/s.

**Table 4:** Prognostic performances of grey matter-to-white matter ratios in brain CT for predicting three-month poor neurological outcome.

Characteristics	AUC (95% CI)	*p*-Value	Sensitivity (%)	Specificity (%)	PPV	NPV
CN/CC	0.705 (0.568–0.819)	0.003	33.3	100.0	100.0	51.1
P/CC	0.652 (0.513–0.775)	0.048	6.06	100.0	100.0	42.6
T/CC	0.692 (0.554–0.808)	0.007	18.18	100.0	100.0	46.0
CN/PIC	0.607 (0.468–0.735)	0.161	12.12	100.0	100.0	44.2
P/PIC	0.559 (0.420–0.691)	0.464	3.0	100.0	100.0	41.8
T/PIC	0.588 (0.448–0.718)	0.254	6.06	100.0	100.0	42.6
Average GWR	0.687 (0.549–0.804)	0.01	18.18	100.0	100.0	46.0
Average (CC) *	0.719 (0.583–0.831)	0.002	18.18	100.0	100.0	46.0

CN, caudate nucleus; P, putamen; T, thalamus; PIC, posterior limb of internal capsule; CC, corpus callosum; *, average of CN/CC, P/CC, and T/CC.
